# Perceptual Response Training for Reduction of Injury Risk Among High School Girls’ Soccer Players

**DOI:** 10.3390/brainsci14111091

**Published:** 2024-10-30

**Authors:** Gary B. Wilkerson, Kyle S. Mether, Zoë A. Perrin, Samuel L. Emberton, Lynette M. Carlson, Jennifer A. Hogg, Shellie N. Acocello

**Affiliations:** 1 Department of Health & Human Performance, University of Tennessee at Chattanooga, Chattanooga, TN 37403, USA; lynette-carlson@utc.edu (L.M.C.); jennifer-hogg@utc.edu (J.A.H.); shellie-acocello@utc.edu (S.N.A.); 2 Department of Intercollegiate Athletics, University of Tennessee at Chattanooga, Chattanooga, TN 37403, USA; kyle-mether@utc.edu (K.S.M.); sam-emberton@utc.edu (S.L.E.); 3 Department of Intercollegiate Athletics, Lipscomb University, Nashville, TN 37204, USA; zoeperrin@7perrins.com

**Keywords:** virtual reality, concussion, mild traumatic brain injury, musculoskeletal injury, intra-individual variability, performance enhancement

## Abstract

**Background/Objectives:** Neural processes involved in visual detection, decision-making, and motor plan execution are believed to play a key role in the avoidance of sport-related injuries, but very little evidence exists to guide the development of training activities for the optimization of brain function. Immersive virtual reality provides a means to precisely measure the amount of time that elapses from visual stimulus presentation to the initiation of a motor response (i.e., perceptual latency) or its completion (i.e., response time). **Methods:** The median value of a metric quantifying both the speed and accuracy (i.e., the rate correct per second of response time) of 50 high school female soccer players was used to assign those who exhibited suboptimal performance to a training program. Training sessions required less than 5 min and the number of sessions completed over a 7-week period ranged from 3 to 13 (median = 5). **Results:** Among 42 players available for follow-up assessment at 8 weeks after the first practice session (training n = 19; comparison n = 23), the results of regression-discontinuity analyses demonstrated statistically significant differences (*p* < 0.05) for metrics representing fast/accurate movement initiation (i.e., the rate correct score for perceptual latency, *p* = 0.016) and across-trial consistency (i.e., perceptual latency variability, *p* = 0.027). From the first practice session to the end of the soccer season, 12 injuries were sustained by 10 players (four concussions and eight musculoskeletal injuries). A time-to-event analysis demonstrated strong associations with perceptual latency variability ≥ 0.143 (Hazard Ratio = 15.43, *p* = 0.011) and a lifetime history of at least one concussion (Hazard Ratio = 8.84, *p* = 0.008). **Conclusions:** The strong association of movement initiation consistency with the avoidance of concussion or musculoskeletal injury suggests that the training program may have a highly beneficial far-transfer effect.

## 1. Introduction

Among all injuries sustained by high school female soccer players over a nine-year period, 68% were lower extremity musculoskeletal injuries and 22% were concussions [1]. Repetitive occurrences are common for both injury types, and concussion is known to elevate the risk for subsequent musculoskeletal injury among female high school soccer players [2,3]. The exact mechanism by which concussion increases the susceptibility to musculoskeletal injury has not yet been definitively established [2,4], but prolonged disruption of integrative neural processes involved in visual detection, decision-making, and motor plan execution is believed to play a key role [5,6,7,8]. Because the cumulative effects of multiple concussions or multiple musculoskeletal injuries can impose performance limitations and long-term adverse effects on quality of life, improved methods are needed to identify and address potentially modifiable risk factors.

Soccer has the highest rate of concussion occurrence among female high school sports at 6.11 per 10,000 athlete-exposures [9]. Despite abundant evidence that a concussion is equivalent to a mild traumatic brain injury (mTBI), the former term is widely interpreted as a designation for a relatively benign condition that produces temporary symptoms [10]. The typical resolution of clinical symptoms within one to two weeks after concussion is inconsistent with evidence of both acute and long-term abnormalities in brain microstructure and function [11,12,13]. A substantial body of evidence suggests that a delayed and clinically silent neuroinflammatory process can increase susceptibility to subsequent concussion and cumulative damage [14,15,16]. The insufficient sensitivity of most clinical tests in detecting subtle post-concussion effects might be attributable to the compensatory activation of supplemental neural resources to a level that is adequate to meet task demands [11,14], thereby masking a diminished capability to meet the demands of high-intensity functional activities.

Dual-task tests that require the simultaneous performance of a cognitive task and a motor task appear to provide greater sensitivity than single-task tests [17,18], but the lack of a common goal for the respective tasks may present an important limitation. An immersive virtual reality (VR) test that imposes a demand for integrated visual, cognitive, and motor processing among the primary visual cortex, middle temporal area, lateral intraparietal area, frontal eye fields, basal ganglia, superior colliculus, dorsolateral prefrontal cortex, anterior cingulate cortex, subthalamic nucleus, supplementary motor area, and premotor cortex, as well as providing measurements of perceptual–motor behavioral performance, appears to provide very good discrimination between individuals who self-report a history of at least one concussion in their lifetime and control participants who deny ever having sustained a concussion [19]. Performance metrics derived from VR testing have been prospectively associated with the occurrence of a strain or sprain affecting the core (i.e., the lower back or abdomen) or lower extremity [20], which are particularly susceptible to delayed perceptual–motor responsiveness to sensory inputs associated with rapidly changing displacements of body mass, and ground reaction forces.

Furthermore, relatively brief VR training sessions have been documented to result in significant improvements in performance metrics for college wrestlers with a history of concussion [21]. An important implication of the collective results of these studies is the potential for VR assessment of perceptual–motor efficiency to identify individual athletes who might derive benefit from targeted training for risk mitigation.

Currently, sport-related concussion management guidelines do not include recommended procedures for the rehabilitation of post-acute dysfunction or training to enhance resilience to subsequent injury [22]. The lack of sufficient research evidence to support a specific approach [23,24] may be due to a combination of limitations imposed by the study design, the statistical model, and an incomplete understanding of relationships between neural processes and behavioral responses. Randomized assignment to either an experimental group or a control group is not generally acceptable within a competitive sports program due to the concern that an athlete with elevated injury risk would be deprived of a potentially beneficial activity. An alternative approach is provided by the regression-discontinuity study design [25], which creates groups on the basis of a performance metric that assigns individuals who are likely to derive the greatest benefit to a training program (e.g., those with a performance value below the median for all athletes on the team). In the context of training for the improvement of alternative responses, perceptual–motor efficiency may be best represented by a performance metric that incorporates both accuracy and speed [21]. Arguably, the most important transfer of training effect is a difference in injury incidence between groups of athletes that is large enough to support a counterfactual explanation for the likely results of providing versus withholding a specific preventive intervention [26].

Reductionist research methods appear to offer limited insights that will advance the understanding of a complex system that can produce dynamic change in injury susceptibility over time [27,28]. Although observational research conducted in a real-world setting can rarely achieve a high level of control over extraneous factors, incorporating study procedures into a competitive team’s schedule of activities is essential for successful implementation. For example, practical considerations dictated that players participating in the immersive VR training were simultaneously exposed to the potential for injury occurrence. Time-dependent analyses of exposure–outcome associations provide a means to accommodate such a scenario [29,30], and group-specific analyses restricted to different time periods may provide plausible counterfactual support for an intervention that might have resulted in different outcomes [26]. Despite the research challenges presented by the realities of a competitive sports setting, exploratory statistical modeling has the potential to provide beneficial insights [31]. Novel elements of this research included moving visual stimuli that imposed a series of two alternative forced-choice responses; a requirement for coordinated neck rotation, arm reaching, and whole-body single-step lunges in a direction corresponding to stimulus features; and the quantification of elapsed time for both the initiation of the body movements and completion of the response. Furthermore, few studies have examined the potential benefit of providing a training program for athletes whose baseline performance level may be associated with elevated injury risk. Thus, this study was designed to evaluate a hypothesized benefit of immersive VR training on the incidence of sport-related injuries among high school female soccer players over the course of a pre-season practice period and a subsequent full season of competitive events.

## 2. Materials and Methods

### 2.1. Participants

A collaboration among three private high school athletic programs yielded a total of 50 female soccer players (15.2 ± 1.2 years, 1.65 ± 0.06 m, 56.9 ± 6.7 kg) who volunteered to participate in baseline and follow-up immersive VR assessments, as well as intervening training sessions for the remediation of suboptimal baseline performance. All procedures were approved by the Institutional Review Board of the University of Tennessee at Chattanooga, including the documentation of parent or guardian informed consent and minor athlete assent. The only exclusionary criterion was an injury-related impairment that limited the ability to perform rapid single-step lunging movements. Data collected from players who discontinued soccer participation at any point in time, declined participation in the training program, or chose not to participate in the follow-up assessment were discarded.

### 2.2. Procedures

**Figure 1:**
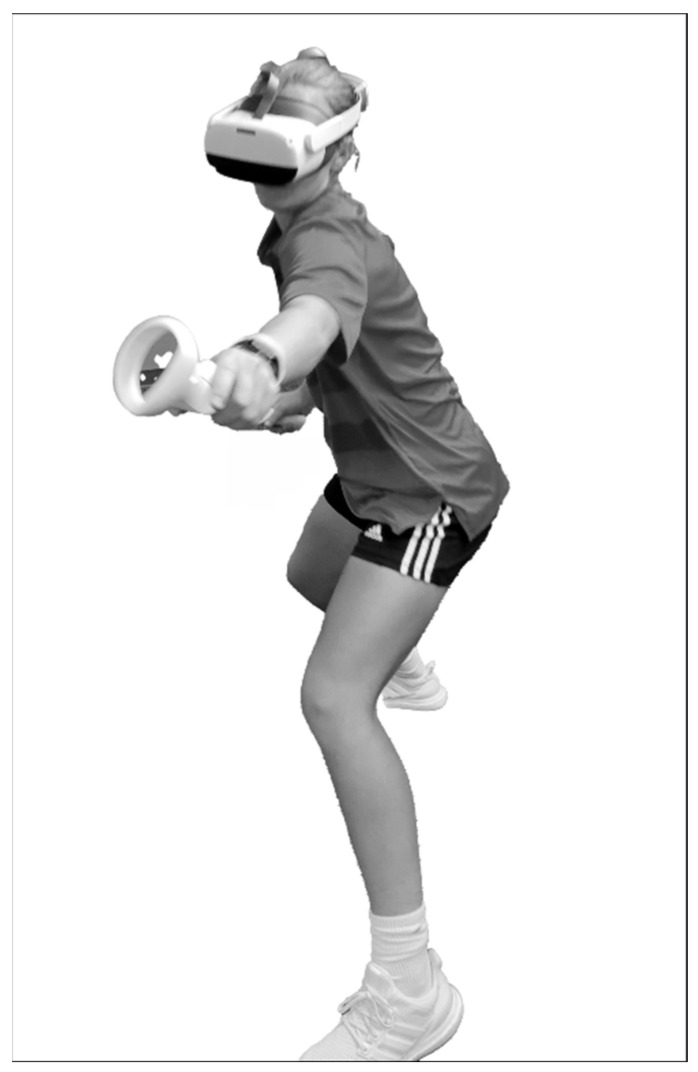
Simultaneous reaching and lunging movement to make virtual hand controller contact with a response target for a visual stimulus moving horizontally across the headset display. (Figure reproduced with permission from Ref. [20].

Approximately four weeks prior to the start of pre-season practice sessions (range of 27–28 days), each of the athletes completed a 40-trial immersive VR test that required simultaneous reaching and lunging responses to visual stimuli moving horizontally across a headset display (Figure 1). The visual stimuli included a white circle or a white ring that initially appeared in either a central or a peripheral position and moved horizontally across a black background. The visual stimulus–response instructions were to locate and make hand controller contact with a response target (a spherical object) positioned beyond the athlete’s arm reach distance on either the right or left side. The “correct” response to a white circle was to contact the response target located in the direction corresponding to that of the circle’s horizontal movement. The “correct” response to a white ring was to contact the response target located in the direction opposite to that of the ring’s horizontal movement. No feedback was provided to indicate whether a given response was correct or incorrect. Further details of the instrumentation and test procedures are provided in previous publications [19,20,21].

The response time (RT) was defined as the amount of time that elapsed between the appearance of a visual stimulus and the instant of maximum horizontal displacement of a body segment toward a virtual response target. We defined perceptual latency (PL) as the portion of the RT required to detect, identify, and initiate a response to a visual stimulus, which corresponded to 10 cm of linear displacement (i.e., an arm reach and a lunge step) or 6° of angular displacement (i.e., neck rotation). Movement time (MT) was defined as the difference between the RT and PL (i.e., RT = PL + MT). The inherent trade-off between the response speed and accuracy was captured by the calculation of the rate correct per second (RCS) score [32], which was derived from the number of correct responses divided by the sum of the 40 RT values (RCS-RT), or the sum of the 40 PL values (RCS-PL), for hand controller movements (i.e., arm reaching). The intra-individual standard deviation of RT and PL values for all 40 trials defined RT variability (RTV) and PL variability (PLV) for neck rotation, arm reaching, and lunge step movements.

We used a regression-discontinuity study design that assigned the 50 participants to either a training group (Train: n = 25) or a comparison group (Comp: n = 25) by a baseline performance cut point (i.e., median value) for RCS-RT. The initiation of a VR perceptual response training (PRT) program coincided with the date of the first allowable organized team activity. The availability of PRT was terminated in the third week of the regular competitive season (53 days after its initial availability). The 25 athletes assigned to the program participated in soccer at three different private high schools, each of which had differing activity schedules. An athletic trainer who served as program coordinator for a given team administered the PRT sessions at whatever times the training group players were available.

The stimulus–response instructions for PRT were identical to those for the baseline assessment, but the immersive VR incorporated numerous game-like visual and auditory elements designed to optimize user engagement (e.g., background music, a futuristic environment, and sounds designating correct versus incorrect responses). Each PRT session consisted of 2 sets of 20 trials, which required less than 5 min to complete. The VR program was automated to progress each individual athlete to an increased level of task difficulty whenever ≥90% (18/20) correct responses were recorded. The first of 6 difficulty levels presented the onset of each moving visual stimulus (i.e., a circle or ring) at the center of the headset display, whereas the second level presented their initial location at either the center or the periphery of the visual field. The third level introduced 2 distractors (i.e., circles or rings) located above and below the target stimulus in a column. Any of the 3 stimuli could move horizontally in either a right or left direction, and the centrally located target stimulus flashed on and off to facilitate its detection. At the fourth level, any of the 3 stimuli could initially appear in a center or a peripheral location on the display and any one of them could be designated as the target stimulus by flashing on and off. The fifth level increased the horizontal movement speed of the 3 stimuli and the sixth level presented 2 distractor stimuli flashing at a lower frequency than a target stimulus flashing at a higher frequency.

All injuries sustained between the first pre-season practice session and the end of a team’s final game (including playoff games) were documented by an athletic trainer, with injury defined as any concussion or core or lower extremity (CLE) strain or sprain that interrupted participation in a practice session or game and that resulted in treatment or some degree of subsequent physical limitation. The injury surveillance period for a given athlete was divided into 2 phases: Phase 1 was defined as the interval from the first pre-season practice session to the date of the follow-up immersive VR assessment, and Phase 2 was defined as the interval from the latter time point to the end of the last game (including playoff games). The follow-up immersive VR assessment procedures were identical to those used for the baseline assessment.

### 2.3. Data Analysis

Regression-discontinuity analysis was used to identify any changes in performance capabilities that could be confidently attributed to participation in the PRT program. Comparisons of change among various performance metrics were further assessed by repeated measures analysis of variance group X session interaction effect size (η_p_^2^) and statistical power (1 − β). Any analysis result affected by a positive distributional skew (i.e., Shapiro–Wilk test *p* < 0.05) was repeated after the natural logarithmic transformation of the data, with back-transformation of natural log mean values used to estimate the geometric mean of the original data. Phase-dependent receiver operating characteristic (ROC) analyses were used to assess exposure–outcome associations, with the determination of area under curve (AUC) values and the use of Youden’s Index for the identification of a cut point for maximum discrimination. Baseline performance metrics were assessed for a Phase 1 positive predictive value (PPV) and negative predictive value (NPV), whereas follow-up performance metrics were assessed for a Phase 2 PPV and NPV. Cox regression analysis was used to identify the strongest predictors of injury hazard across the course of the entire surveillance period, and a Kaplan–Meier time-to-event graph was generated for the depiction of comparative effects on injury occurrences. The most recent performance level measured prior to injury occurrence (i.e., baseline or follow-up) was used for both the Cox regression and Kaplan–Meier procedures. All available data, including a lifetime history of concussion, the occurrence of a CLE injury during the 12-month period preceding the first practice session, and the number of games played, was analyzed to discover the strongest predictors of subsequent injury. Because this exploratory study was not designed to deductively test null hypotheses, no multiple comparisons correction was made to an alpha level of 0.05 for any of the test results [33]. All analyses utilized IBM SPSS Version 29.0 (IBM Corp., Armonk, NY, USA).

## 3. Results

**Figure 2:**
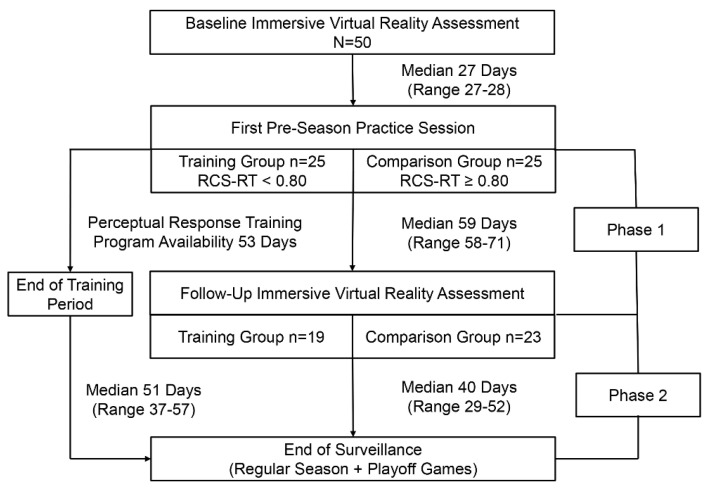
A flowchart depicting the time courses of study events, cases lost to follow-up in both the training and comparison groups, and injury surveillance phases.

**Figure 3:**
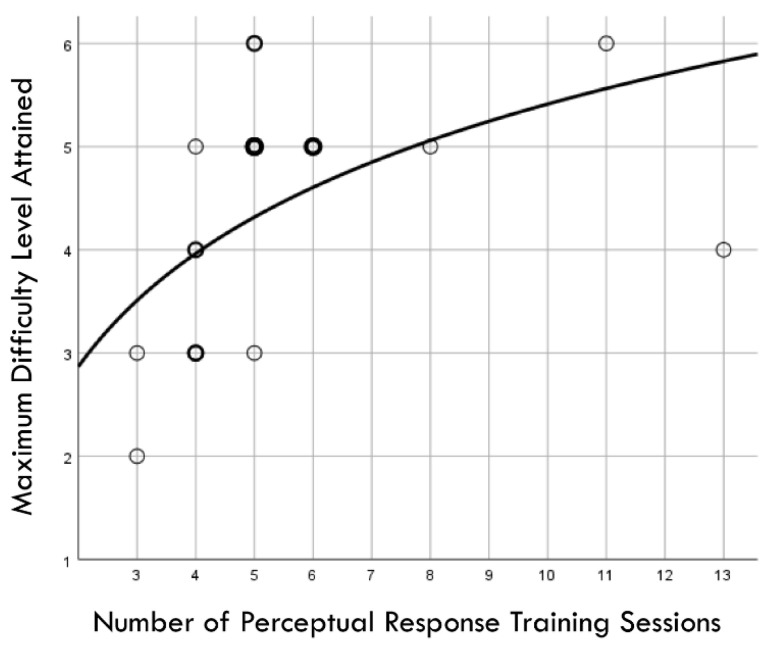
Graphic depiction of a logarithmic relationship between the number of perceptual response training sessions completed and the maximum difficulty level achieved by 19 high school female soccer players. Light circle boundaries represent a single case, moderately thicker/darker circle boundaries represent 2 cases, and thickest/darkest circle boundaries represent 3 cases.

Figure 2 presents a flowchart depicting the time courses of study events, cases lost to follow-up in both the training and comparison groups, and injury surveillance phases. The median value for RCS-RT was 0.80 (range: 0.32 to 1.25), and this was used to create two groups of 25 players each (i.e., <0.80 versus ≥0.80). Discontinued participation in soccer for personal reasons or absence from a scheduled follow-up assessment resulted in the loss of six players assigned to the training group and two players assigned to the comparison group. Among 19 players who completed the training program, the median number of sessions completed was five (range: 3 to 13). The number of training sessions completed demonstrated a logarithmic relationship with the maximum PRT difficulty level achieved (r^2^ = 0.291, *p* = 0.017; Figure 3).

**Figure 4:**
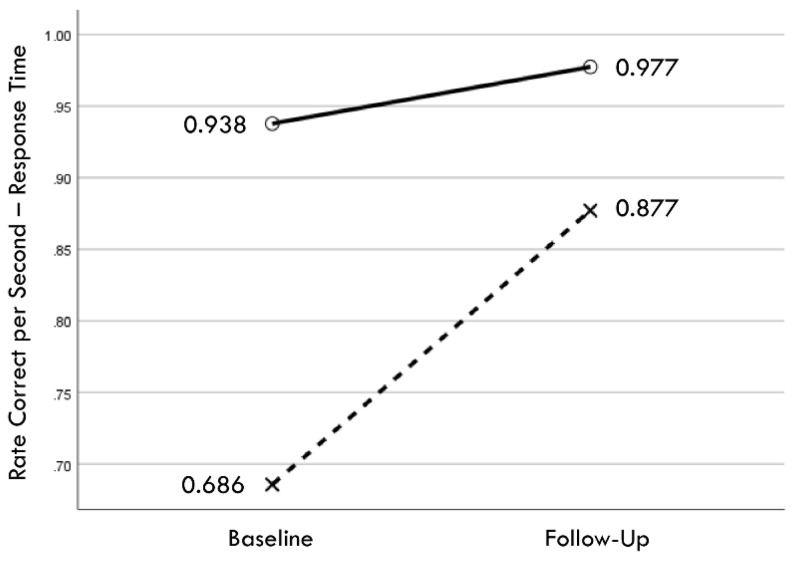
Change in mean values from baseline to follow-up assessments of rate correct per second of response time for training (×) and comparison (○) groups.

**Figure 5:**
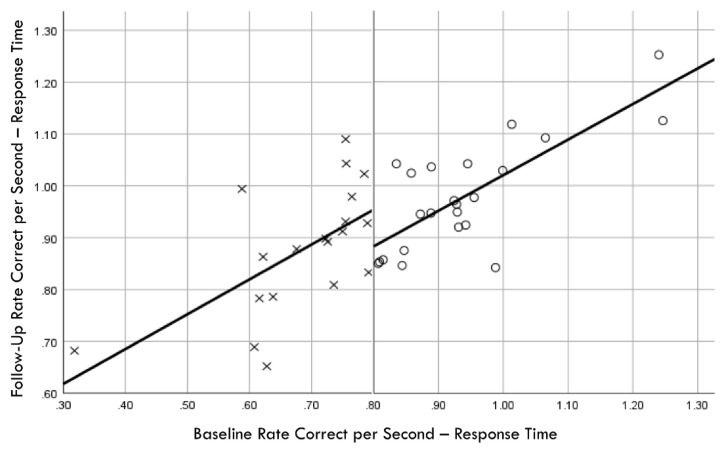
Baseline rate correct per second of response time correlation with follow-up rate correct per second of response time; regression discontinuity evident at cut point for training (×) and comparison (○) groups.

**Figure 6:**
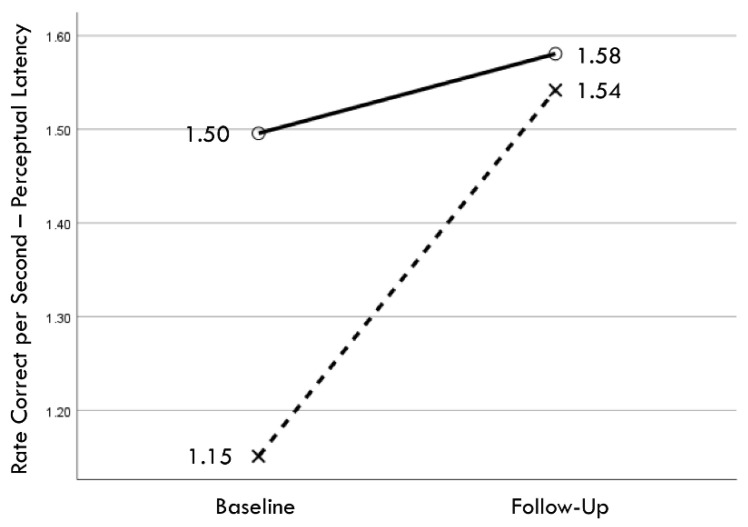
Change in mean values from baseline to follow-up assessments of rate correct per second of perceptual latency for training (×) and comparison (○) groups.

**Figure 7:**
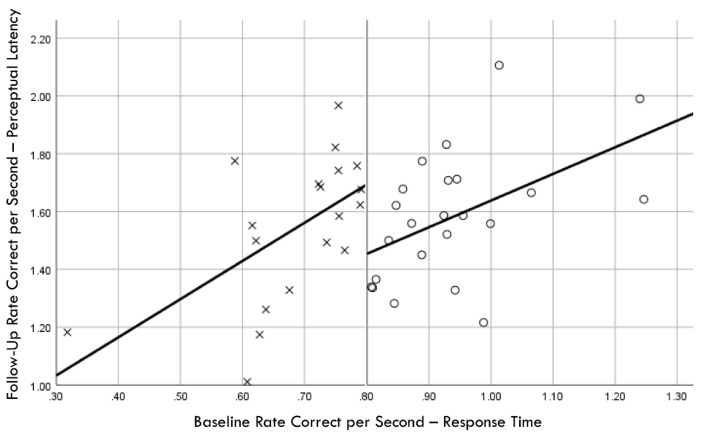
Baseline rate correct per second of response time correlation with follow-up rate correct per second of perceptual latency; regression discontinuity evident at cut point for training (×) and comparison (○) groups.

**Figure 8:**
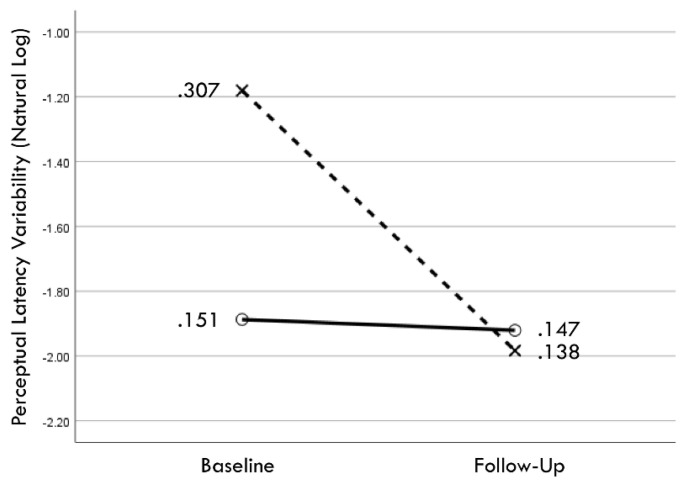
Change in geometric mean values (i.e., back-transformation of natural logarithm mean values) from baseline to follow-up assessments of perceptual latency variability for training (×) and comparison (○) groups.

**Figure 9:**
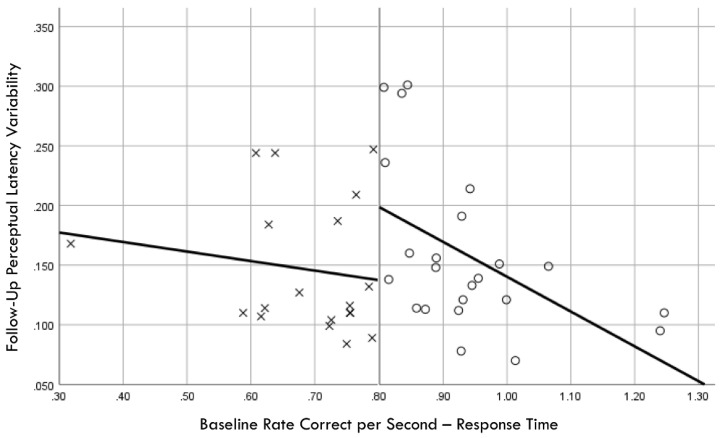
Baseline rate correct per second of response time correlation with follow-up perceptual latency variability; regression discontinuity evident at cut point for training (×) and comparison (○) groups.

Distribution normality was confirmed for both the baseline and follow-up RCS-RT data, which demonstrated average improvements in the training and comparison groups of 28% and 4%, respectively (group X session *p* < 0.001, η_p_^2^ = 0.42, 1 − β = 1.00; Figure 4). Regression-discontinuity analysis confirmed that a linear model provided the best fit to the data (i.e., quadratic, quadratic interaction, and linear interaction terms were not significant), but the group effect at the cut point was not statistically significant (beta coefficient = 0.071, *p* = 0.075; Figure 5). The analysis was repeated for the RCS-PL metric, which demonstrated distributional normality and average improvements in the training and comparison groups of 34% and 5%, respectively (group X session *p* < 0.001, η_p_^2^ = 0.31, 1 − β = 0.99; Figure 6). Regression-discontinuity analysis confirmed that a linear model provided the best fit to the data, with a statistically significant group effect at the cut point (beta coefficient = 0.236, *p* = 0.016; Figure 7). Both baseline and follow-up data for PLV demonstrated a strong positive distribution skew (Shapiro–Wilk *p* < 0.001), which were not significantly different from normality after natural logarithmic transformation (Shapiro–Wilk *p* = 0.161 and *p* = 0.111, respectively). Based on geometric means derived from back-transformation of the natural logarithmic data, the average reductions in PLV in the training and comparison groups were 55% and 3%, respectively (group X session *p* = 0.004, η_p_^2^ = 0.19, 1 − β = 0.85; Figure 8). Regression-discontinuity analyses of both the original and log-transformed PLV data confirmed that a linear model provided the best fit, with similar group effect levels of statistical significance for the log-transformed data (beta coefficient = −0.384, *p* = 0.025) and the original data (beta coefficient = −0.063, *p* = 0.027; Figure 9).

A total of 10 injuries were sustained by eight players who completed the follow-up assessment, which included four concussions, two ankle sprains, one knee sprain, one groin strain, and two low back strains. Both players who sustained a second injury had first sustained a concussion. Time-dependent ROC analyses of Phase 2 exposure–outcome associations for injuries sustained by 12% (5/42) of the players identified optimal cut points of RCS-PL ≤ 1.53 (AUC = 0.605, 24% PPV, 96% NPV) and PLV ≥ 0.143 (AUC = 0.676, 21% PPV, 96% NPV). Only one player in the training group sustained a Phase 1 injury, but an ROC analysis of Phase 1 injuries sustained by 17% (4/23) of players in the comparison group also identified a PLV ≥ 0.143 cut point (AUC = 0.559, 27% PPV, 92% NPV) for optimal discrimination between injured and non-injured players.

**Figure 10:**
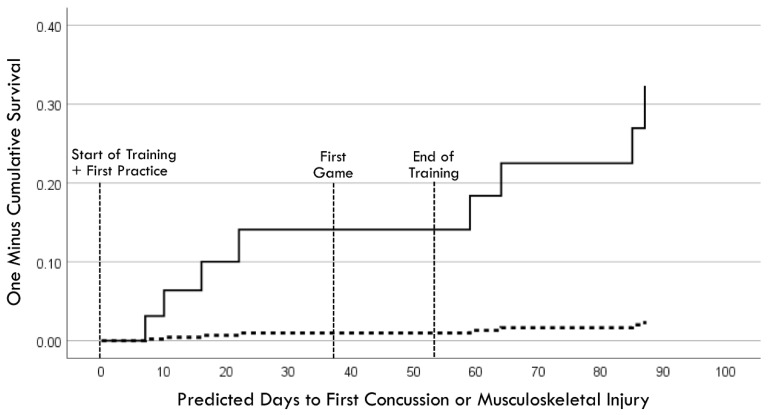
Cox regression prediction model of time to first injury occurrence for pre-injury perceptual latency variability ≥ 0.143 (solid line) versus <0.143 (dashed line), with adjustment for lifetime history of concussion.

Separate univariable Cox regression time-to-event models for the entire surveillance period (i.e., Phases 1 and 2) used the pre-injury performance measurement acquired in the closest temporal proximity to a player’s first injury, whereas the follow-up performance measurement was used for each of the non-injured players. The entry of continuous performance measurements demonstrated a stronger effect for RCS-PL (Model χ^2^ [1] = 7.59, *p* = 0.006) compared to PLV (Model χ^2^ [1] = 5.16, *p* = 0.023). Cox regression was also used to compare Hazard Ratio (HR) values for binary variables, which included a lifetime history of concussion (HR = 5.54, *p* = 0.032), a history of CLE injury during the 12 months prior to baseline testing (HR = 4.36, *p* = 0.079), and high exposure to injury risk based on participation in ≥19 games (HR = 3.02, *p* = 0.137). Entry of the performance measures as binary variables demonstrated very similar effects for RCS-PL ≤ 1.53 (HR = 10.71, *p* = 0.027) and PLV ≥ 0.143 (HR = 10.09, *p* = 0.031). Backward stepwise Cox regression analysis of binary variables identified PLV ≥ 0.143 (adjusted HR = 15.43, *p* = 0.011) and a lifetime history of concussion (adjusted HR = 8.84, *p* = 0.008) as the combination of factors yielding the strongest association (model χ^2^ [2] = 15.55, *p* < 0.001; Figure 10). There was an 83% (5/6) incidence of injury for players with both factors. Among players with only one of the two factors, the injury incidence was 9% (2/23) with pre-injury PLV ≥ 0.143, and 4% (1/23) with a lifetime history of concussion. There were no injuries among the 13 players who had neither factor (χ^2^ [2] = 19.68, *p* < 0.001).

**Figure 11:**
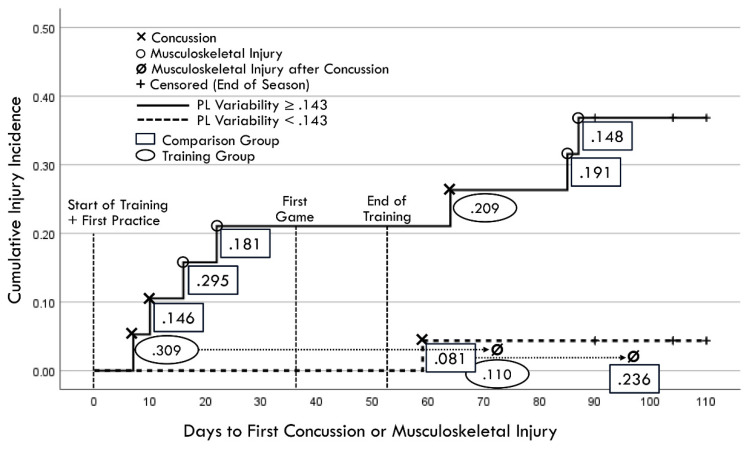
Kaplan–Meier depiction of actual injury occurrences (annotated to include second injuries identified by dotted lines and arrows projected from first injury occurrences). Rectangles identify comparison group cases and ovals identify training group cases, with values representing pre-injury perceptual latency variability measured closest to injury day).

**Figure 12:**
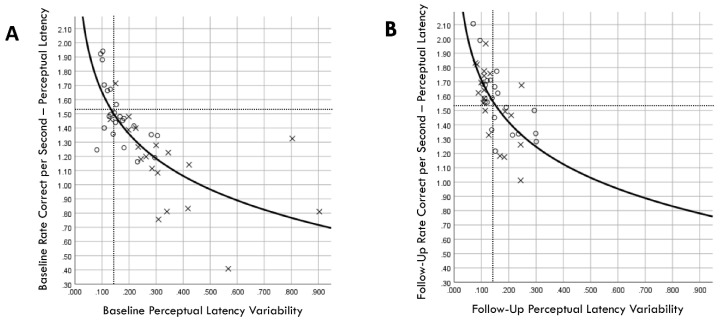
Correlation between rate correct per second of perceptual latency and perceptual latency variability for training (×) and comparison (○) groups at baseline (**A**) and follow-up (**B**) assessments. Dashed lines correspond to cut points derived from receiver operating characteristic analyses for optimal discrimination between between injured and non-injured players (favorable RCS-PL > 1.53 and favorable PLV < 0.143).

A Kaplan–Meier graphic depiction of actual injury occurrences was annotated with designations of comparison group versus training group membership and pre-injury PLV values (Figure 11). Because RCS-PL and PLV demonstrated very similar Cox regression results for both continuous and binary analyses, bivariate relationships between the continuous variables were assessed for both the baseline and follow-up results (Figure 12). A single player assigned to the training group demonstrated a favorable baseline RCS-PL > 1.53, whereas 58% (11/19) exceeded that value at the post-training follow-up. Similarly, only one player assigned to the training group demonstrated a favorable baseline PLV < 0.143, but 63% (12/19) demonstrated across-trial variability below that value at the follow-up.

## 4. Discussion

Our sequential exploratory approach and time-dependent analysis of the data fulfilled the study purpose by yielding results that appear to be highly relevant to the potential for a reduction in injury risk among high school female soccer players, particularly among those with a history of concussion. Prior to study initiation, the RCS-RT metric was chosen to represent the overall speed and accuracy in the performance of a task designed to require time-constrained integration of perceptual, cognitive, and motor processes. After the assignment of participants to training and comparison groups based on low versus high baseline RCS-RT values, subsequent analyses of performance improvements attributable to the PRT program demonstrated changes of the greatest magnitude for RCS-PL and PLV. Thus, the apparent training adaptation occurred in “perceptual and cognitive” processes that preceded the initiation of a movement response to a greater extent than the neuromuscular component of the task. This finding may explain the lack of lower extremity injury risk reduction recently reported for female adolescent athletes with a history of concussion following the completion of a 6-week neuromuscular training program that did not include time-constrained perception and decision-making components [34].

Despite the importance of neuromuscular control for injury avoidance [35,36], the findings of our study are consistent with those of previous research suggesting that perceptual and decision-making processes play a more important role than motor processes in producing rapid responses to dynamic visual stimuli [37,38]. Thus, an understanding of brain–behavior relationships is essential for the design and implementation of strategies for a reduction in both primary and secondary injury risk [39,40]. The “drift-diffusion” model of decision-making provides a mathematical framework that relates latent cognitive processes to observable actions [41,42,43], as well as structural and functional impairments associated with brain disorders [44]. The term “drift” refers to the speed of information processing that leads to the accumulation of evidence to support a decision, whereas the term “diffusion” refers to scattered background noise created by stochastic spike discharges of neurons and fluctuating neural membrane potentials during the evidence accumulation process [38]. A combination of deterministic and random processes affects both brain signal variability and behavioral performance variability [45], such that identical stimuli can produce variable responses [41,46]. Interrelated neural processes that accumulate sensory evidence and set a decision threshold ultimately determine the speed, accuracy, and consistency of successive responses to external stimuli [38,47,48,49], which may be impaired by the post-concussion development of focal axon swellings that interrupt the transmission of neural signals [50].

Experience-related neuroplasticity within circuits connected to the anterior cingulate cortex [51,52] and the subthalamic nucleus [44,47] can increase or decrease the threshold for a sufficient reduction in decision uncertainty [41]. A high threshold reduces response errors but delays the generation of motor commands by the superior colliculus [49] and premotor cortex [38]. Conversely, a low threshold produces faster response initiation at the cost of a higher error rate. This speed–accuracy tradeoff is represented by the RCS performance metric, which incorporates both aspects of the decision-making process. The rate of evidence accumulation is dependent on a somewhat separable mechanism relating to the efficient encoding and transmission of sensory inputs that minimizes noise interference [41]. Visual inputs conveyed by the optic nerve to the visual cortex are transmitted to the middle temporal area and the lateral intraparietal sulcus of the posterior parietal cortex [39], where they are integrated with intrinsically generated brain signals that fluctuate from moment to moment [38,48]. Although repeated presentations of an identical visual stimulus will inevitably result in some amount of response variability from trial to trial [45], modulations of the amplitude, phase, and frequency of neural oscillations can optimize the speed, accuracy, and consistency of successive responses [53,54]. Thus, our finding of a substantial and statistically significant inverse correlation between RCS-PL and PLV suggests that they represent somewhat different aspects of related neural processes. Furthermore, significant post-training improvements were evident for both performance metrics.

## 5. Study Limitations

The limitations of this study included inconsistency in the number and frequency of PRT sessions among athletes assigned to the training program, along with a relatively low number of injury occurrences. Some unknown number of false-positive classifications may have resulted from participant failure to accurately report their concussion history, or the effects of repetitive head impacts that did not produce acute concussion symptoms [55]. Concussion has been found to affect both brain connectivity and lower extremity motor control [56], but our observational study design simply demonstrated strong associations of suboptimal PLV and concussion history with injury occurrences. Whether high PLV was a consequence of concussion history or a condition that existed prior to a past concussion is unknown. Despite these acknowledged limitations, our findings support the potential value of immersive VR training for the improvement of time-constrained visual perception and decision-making, which may be essential for injury avoidance.

Although the results of our analyses suggest that the speed, accuracy, and consistency of perceptual–cognitive processes that initiate movement play the key role in the overall perceptual–motor response to visual stimuli (i.e., RCS-PL and PLV), the integration of time-constrained visual detection and decision-making with whole-body motor responses may be essential for transferability of training effects to performance in a competitive sport environment [57]. Future hypothesis-driven studies with large sample sizes are needed to definitively document causal relationships that may exist among mild traumatic brain injury, impaired brain processing of visual stimuli, and elevated risk for sport-related concussion and musculoskeletal injury [58]. Our findings may or may not extend to other sex- and sport-specific athlete populations, and the combination of frequency and duration of PRT sessions required to achieve an optimal training effect is currently unknown. The are no known adverse effects associated with PRT, but widespread implementation will require more evidence of its beneficial far-transfer effects on sports performance and injury avoidance.

## 6. Conclusions

Our exploratory approach to the analysis of observational data provided compelling evidence that supports a specific approach to the optimization of time-constrained visual perception and decision-making, which further appears to reduce sport-related injury risk among female high school soccer players. Neuromuscular training has clearly been shown to reduce injury risk, but training-induced adaptations in brain processes that initiate body movements may be equally or more important for athletes who exhibit suboptimal perceptual responses to dynamic visual stimuli. Because the study findings closely align with the characteristics of the well-established drift-diffusion model of decision-making, we believe they substantially advance the understanding of brain–behavior relationships needed for further development of effective injury prevention strategies.

## Data Availability

The data presented in this study are available from the corresponding author upon institutional approval. The data are not publicly available, due to an institutional restriction on the release of data acquired from minor participants. A specific request from an individual who possesses research credentials must be reviewed and approved.
